# Global health and human well-being – A systematic review

**DOI:** 10.3934/publichealth.2025019

**Published:** 2025-03-13

**Authors:** Amir Khorram-Manesh, Lesley Gray

**Affiliations:** 1 Department of Surgery, Institute of Clinical Sciences, Sahlgrenska Academy, Gothenburg University, 413 45, Gothenburg, Sweden; 2 Center for Disaster Medicine, Gothenburg University, 405 30, Gothenburg, Sweden; 3 Gothenburg Emergency Medicine Research Group (GEMREG), Sahlgrenska University Hospital, 413 45, Gothenburg, Sweden; 4 Department of Primary Health Care & General Practice, University of Otago, Wellington, 6242, New Zealand

**Keywords:** equity, global, heath, human, well-being, sustainable development goals

## Abstract

Global health aims to improve health outcomes and promote equity by addressing transnational health issues through cross-disciplinary collaboration. This field merges preventive measures with clinical care to enhance health and reduce disparities. On the other hand, human well-being includes material and non-material factors that influence quality of life and personal fulfillment. Together, they are crucial for sustainable development, aligning with the UN's Sustainable Development Goals. Though often examined separately, understanding their interconnectedness can reveal the root causes of global challenges, such as pandemics and climate change, and inform comprehensive strategies for a healthier, more equitable world. This systematic review updates the current challenges and opportunities in global health and well-being. It highlights the importance of scalable, cost-effective solutions, incorporating global health, environmental sustainability, and local contexts to address issues like antimicrobial resistance (AMR), zoonotic diseases, and climate-related health impacts. The study advocates for a multidisciplinary approach, integrating knowledge from medical microbiology, agriculture, environmental science, and traditional practices. Effective solutions should be holistic and inclusive, incorporating bioinformatics in AMR, One Health strategies, and sustainable food systems through regenerative agriculture. These recommendations support broader health equity goals, emphasizing the deep connections among human, animal, and environmental health that are essential for global well-being.

## 1. Introduction

Global health is a field dedicated to studying, researching, and practicing efforts, aiming to enhance health and ensure health equity for people across the globe. It focuses on transnational health challenges, their causes, and solutions and integrates various disciplines within and beyond the health sciences, encouraging inter-, intra-, and cross-disciplinary collaborations. It combines population-based preventive strategies with individual-level clinical care [1],[2]. Well-being is defined as a state of existence that satisfies multiple human needs, including material and non-material living conditions, quality of life, and the capacity to pursue personal goals, thrive, and feel fulfilled [3].

Global health and human well-being are closely intertwined concepts. They are both essential to the development and sustainability of societies worldwide. Global health addresses the health of populations worldwide, while human well-being includes a broader array of factors that influence an individual's quality of life. Together, they make the necessary pillars for creating a thriving, fair, and sustainable global society [4].

Global health is concerned with improving health outcomes, reducing health disparities, and addressing the determinants of health across countries and regions. It encompasses a wide range of issues, including infectious diseases and non-communicable diseases. Human well-being includes all elements of the UN's Sustainable Development Goals (SDGs), which, in harmony and together with global collaboration, can result in improved quality of life worldwide [5],[6].

The academic literature has extensively explored various perspectives on global health and human well-being, with each topic often addressed separately. While ample research provides detailed overviews of global health and human well-being individually, comprehensive discussions examining the combined challenges of these two areas remain limited. This gap in the literature highlights the need for an integrated overview that explores the interconnections between global health and human well-being and the shared challenges they face.

Such an integrated analysis is critical because it will offer a holistic understanding of the relationship between these concepts. It would also shed light on the complex pathways in which health and well-being influence one another, particularly in the context of global issues such as pandemics, climate change, economic disparities, and social inequalities. An in-depth examination of both topics in unison could help identify strategies that address the root causes of these challenges, enabling a more comprehensive and effective approach to improving the overall health and well-being of populations worldwide [1],[3],[5],[6].

Additionally, by providing a mutual picture of the relationship between global health and human well-being, such an overview could offer valuable insights into how integrated policies, programs, and interventions can be developed. It would allow policymakers, researchers, and practitioners to improve their understanding of the common obstacles both fields face and to collaborate on innovative solutions that promote global health equity and sustainable improvements in human well-being. Addressing these challenges collectively could lead to considerable progress toward a healthier, more equitable, and sustainable world for all [4],[6].

Differentiating between global and planetary health is crucial for understanding their unique contributions to human well-being. Planetary health focuses on the interdependence of human health and the Earth's ecosystems, emphasizing sustainable interactions to safeguard both humanity and the planet. Unlike public health, which addresses health protection and promotion within systems, and global health, which targets health improvement across populations worldwide, planetary health considers the broader relationship between civilizations and the ecosystems they depend on [7].

As Raffaella Bosurgi from *The Lancet Planetary Health* explains:

“*Planetary health broadens this discussion by looking at the societies, civilizations, and the ecosystems on which they depend. It offers an exciting opportunity to find alternative solutions for a better and more resilient future. It aims not only to investigate the effects of environmental change on human health but also to study the political, economic, and social systems that govern those effects*.”

This perspective highlights the need for integrated approaches to achieve a sustainable and resilient future [7].

This paper aims to present an updated version of the current challenges and opportunities in global health and human well-being.

## 2. Materials and methods

### 2.1. Study design

The study utilized a systematic literature search approach, adhering to the PRISMA (Preferred Reporting Items for Systematic Reviews and Meta-Analyses) guidelines to ensure thoroughness and transparency [8]. The search was conducted across three key databases, namely PubMed, Scopus, and Web of Science, which collectively cover a broad spectrum of scientific literature and provide a robust foundation for finding relevant studies.

### 2.2. Search

#### 2.2.1. Keywords

The search strategy focused on two primary themes: “global health” and “human well-being”. The search terms “global”, “health”, “human”, and “well-being” were applied both independently and in various combinations to explore the literature comprehensively. This approach aimed to capture a wide range of studies on both topics, ensuring the search was inclusive and comprehensive relative to how each theme is defined: global health (health improvement across populations worldwide) and human well-being (defined as a state of existence that satisfies multiple human needs, including material and non-material living conditions, quality of life, and the capacity to pursue personal goals, thrive, and feel fulfilled) [4]–[7].

#### 2.2.2. Strategy

The search utilized the Boolean combination “global health” AND “human wellbeing” across all selected databases. This strategy was designed to maximize the relevance of the search results by targeting studies that address both themes simultaneously. No restrictions were placed on the publication timeframe, allowing for a broad search scope.

#### 2.2.3. Final searching conditions

The following conditions were applied to the final search:

1. Search fields: title and abstract.

2. Phrase search: “global health” AND “human wellbeing/well-being”.

3. Phrase search: “human health” AND “global wellbeing/well-being”.

4. Keywords: global AND health AND human AND wellbeing/well-being.

5. All dates.

6. All source types.

7. Limited to the English language.

### 2.3. Inclusion criteria

The review included all research papers published in English that employed the specified keywords to explore the relationship between “global health” and “human well-being”. Only original research articles published in peer-reviewed journals were eligible. This criterion ensured that the included studies were of high academic quality and contributed new knowledge to the field.

### 2.4. Exclusion criteria

The review excluded book chapters, conference proceedings, letters, notes, and extended abstracts, as these sources typically provide less detailed information than full journal articles. Studies focusing solely on one of the keywords without addressing the interplay between “global health” and “human well-being” were also excluded. Additionally, non-English language studies were not considered to maintain consistency in language and interpretation.

### 2.5. Eligibility

Studies that met the inclusion criteria and passed the full review process were deemed eligible for inclusion in the analysis. This rigorous process ensured that only relevant and high-quality studies were selected.

### 2.6. Review and selection process

Eligible studies were compiled. Initially, the titles of studies and abstracts were transferred to a Word file for the first review. Papers that passed this stage were subjected to a more detailed second review, which involved a comprehensive evaluation of the full text. Both authors reviewed all papers independently on both occasions. Disagreements were resolved by discussion between the authors until a consensus was achieved and the final decision was made.

#### 2.6.1. First review

In this stage, reviewers examined the titles and abstracts of the 84 papers to identify those that potentially met the inclusion criteria. Relevant studies were selected for the second review on the basis of their initial assessment.

#### 2.6.2. Second review

During the second review stage, 30 selected papers were thoroughly analyzed. Data relevant to the study's objectives were extracted and organized for further analysis in the final step (*n* = 7).

### 2.7. Data collection and analysis

The data collected from the reviewed papers included the study's title, author(s), journal name, and publication year. This information was systematically compiled and presented in a table to facilitate a clear and concise summary of the research findings (Table 1).

### 2.8. Content analysis

A conventional content analysis was conducted to examine the presence of specific words, themes, or concepts within the texts. This qualitative analysis aimed to identify patterns and trends related to the study's focus on “global health” and “human well-being” [9]. The content analysis provided insights into how these themes are addressed in the literature and highlighted areas for further research (Table 2).

### 2.9. Assessment of the scientific evidence

The search outcome resulted in several narrative reviews. Therefore, the Scale for the Assessment of Narrative Review Articles (SANRA) was utilized for the assessment of the scientific evidence of each article [10].

### 2.10. Ethical approval

This does not apply to systematic review of already published data.

### 2.11. Study protocol registration

The study protocol was registered at Figshare (https://doi.org/10.6084/m9.figshare.27050260).

## 3. Results

**Figure 1.**
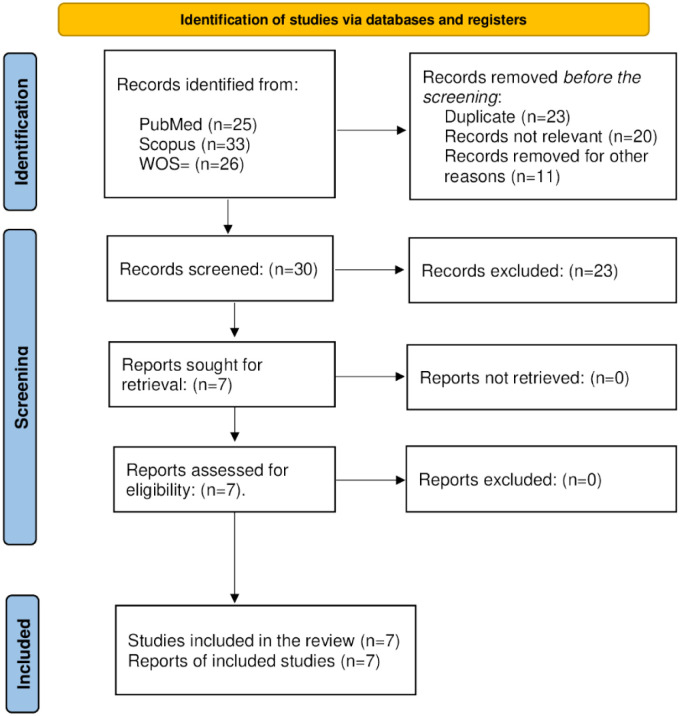
PRISMA 2020 flow diagram for new systematic reviews [7].

The search resulted in the following final hits: PubMed (*n* = 39), Scopus (*n* = 16), and Web of Science (*n* = 13). Figure 1 shows the selection process for the included studies. Seven articles were finally included in this study, unanimously chosen by both authors. Table 1 summarizes key information about each included paper [11]–[17].

In the process of reviewing articles for selection into the final stage, studies on planetary health and One Health were excluded since this paper focused only on the relationship between global health and well-being, targeting health improvement across populations worldwide and examining the topic from a specific global health lens. There were no similarities among the included studies, except the mutual keywords resulting in their inclusion. Nevertheless, each study showed unique content, presenting differences between the included studies. The content analysis resulted in six categories, highlighting important key factors in global health and human well-being.

**Table 1.** The review results of the included papers.

**No.**	**Title**	**Author, Journal, Year**	**Study type**	**Outcome**	**Level of evidence**
**1**	Antimicrobial resistance and its spread is a global threat	Aljeldah, *Antibiotics (Basel)*, 2022 [11]	Narrative review	Antimicrobial resistance (AMR) is a global public health threat with the potential to trigger another pandemic if unchecked. Addressing AMR requires global health solutions based on regional data, promoting positive social norms, behavioral changes, and increased awareness. AMR complicates treatment and challenges current antibiotic guidelines, with resistance linked to complex genetic transmission pathways. Misdiagnosis, broad-spectrum antibiotic use, and slow diagnostics contribute to resistance. However, advancements in DNA sequencing and bioinformatics are improving the real-time detection of AMR, making it a crucial area in medical microbiology.	Low–medium (5/12 p)
**2**	One Health contributions towards more effective and equitable approaches to health in low- and middle-income countries	Cleaveland et al., *Philos Trans R Soc Lond B Biol Sci*, 2017 [12]	Narrative review	Emerging zoonoses with pandemic potential are a global health priority, but endemic zoonoses also significantly affect low-resource settings. While many endemic zoonoses are treatable, timely diagnosis and management are often difficult. Preventive “One Health” interventions, such as animal vaccinations, offer a solution that benefits human and animal health. These interventions are already effective against diseases like rabies, brucellosis, and leptospirosis, which impact human health and livestock in low- and middle-income countries. They argue that One Health approaches can better serve rural communities by fostering trust, community engagement, cross-sector collaboration, strengthening fragile health systems, and aligning global health and sustainability goals.	Medium (6/12 p)
**3**	Climate change adaptation: Where does global health fit in the agenda?	Bowen and Friel, *Global Health*, 2012 [13]	Narrative review	Human-induced climate change will impact most populations in the coming decade, with the poorest and most disadvantaged affected the earliest and most severely. Climate change affects human well-being, safety, and health through both immediate impacts like reduced food yields and storm surges and more complex, indirect pathways involving sectors like water, agriculture, and urban planning. Adaptation efforts, particularly in developing regions with weaker response mechanisms, are gaining attention and funding from global entities like the World Bank and the United Nations. This paper highlights the need to base adaptation on community needs and vulnerabilities, offering opportunities to improve health and reduce inequities through climate adaptation funding.	Medium (7/12 p)
**4**	Food for thought: Making the case for food produced via regenerative agriculture in the battle against non-communicable chronic diseases (NCDs)	Ramkumar et al., *One Health*, 2024 [14]	Narrative review	Non-communicable diseases (NCDs) present a global health challenge, causing significant illness, death, and economic burden. This review highlights the rising incidence of NCDs and explores the role of regenerative agriculture (RA) in mitigating these diseases. It emphasizes the benefits of plant-based diets over those high in processed foods and red meat. It examines how dietary interventions, and a healthy gut microbiome can help disease prevention and management. Notably, the review finds connections between soil and human microbiomes, suggesting that healthier soil from RA practices—characterized by natural inputs, crop rotation, and livestock integration—leads to higher-quality nutrient-rich foods compared with conventional agriculture. With RA's environmental benefits, including carbon sequestration and water conservation, promoting RA-produced foods could enhance human and planetary health. Increasing RA in local food systems may improve food's quality, accessibility, and affordability, contributing to sustainable health and environmental goals.	Medium–high (8/12 p)
**5**	From nature to nanotechnology: The interplay of traditional medicine, green chemistry, and biogenic metallic phyto-nanoparticles in modern healthcare innovation and sustainability	Puri et al., *Biomed Pharmacother*, 2024 [15]	Narrative review	Integrating traditional natural remedies with modern science drives a new era in global healthcare. Natural products, used by 80%–85% of the population worldwide, now demand improved quality, efficacy, and safety. Biogenic phytonanoparticles, created through eco-friendly methods with metals like copper, silver, and zinc, offer sustainable, cost-effective therapies with antioxidant and antiaging benefits. This fusion of traditional knowledge and technology opens promising opportunities for healthcare that prioritizes innovation and sustainability. Metallic nanoparticles, in particular, hold potential across medicine, catalysis, and electronics, paving the way for solutions to today's challenges and advancements for the future.	High (10/12 p)
**6**	Role of circadian clock on the pathogenesis and lifestyle management in non-alcoholic fatty liver disease	Perez-Diaz-del-Campo et al., *Nutrient*, 2022 [16]	Narrative review	Modern lifestyle factors, such as irregular eating patterns and weekly schedules, are key contributors to global health issues like non-alcoholic fatty liver disease (NAFLD). Disrupted circadian rhythms and poor sleep quality can worsen overall health, while social jetlag—caused by mismatches between circadian and social clocks—has been linked to negative metabolic effects. NAFLD management typically focuses on diet and exercise, but circadian preferences and environmental factors should also be considered. Chrononutrition strategies, like intermittent fasting and time-restricted feeding, show promise in aligning behaviors with biological rhythms. However, further research is needed to explore these dietary approaches for NAFLD treatment.	Medium–high (9/12 p)
**7**	The burden of antibiotic resistance of the main microorganisms causing infections in humans–review of the literature	Baciu et al., *J Med Life*, 2024 [17]	Narrative review	Antibiotic resistance is one of the biggest threats to public health, with the potential to trigger another pandemic if left unchecked. This highlights the urgent need for global solutions incorporating data from different regions. Promoting positive social norms, encouraging healthy behaviors, and raising public awareness are key to addressing this issue. Antibiotic resistance complicates treatment, is linked to complex genetic mechanisms, and hampers adherence to existing guidelines. Misdiagnosis, overuse of broad-spectrum antibiotics, and delayed diagnosis also fuel resistance. However, advances in DNA sequencing and bioinformatics are transforming diagnostics, enabling real-time detection of resistance factors, and aiding in developing effective prevention strategies.	Medium–high (9/12 p)

Note: SANRA assessment: High, 9–12 points; medium, 4–8 points; low, 0–4 points.

**Table 2.** The outcome of content analysis shows the key categories impacting global health and human well-being.

Category	Key points	Expanded insights
Antimicrobial resistance (AMR) and global health threats [11],[17]	∘ AMR poses a significant threat to global public health and has the potential to trigger a pandemic if not addressed. ∘ Solutions require regional data and strategies, emphasizing the promotion of social norms and behavioral changes and raising public awareness. ∘ The genetic complexity of AMR, including the transfer of resistance genes between microorganisms, makes it a difficult clinical challenge. ∘ Misdiagnosis, overuse of broad-spectrum antibiotics, and slow diagnostics contribute to resistance development. ∘ Advancements in DNA sequencing and bioinformatics offer real-time detection, enhancing the ability to manage and prevent AMR.	AMR is among the top 10 global health threats, according to WHO. The misuse of antibiotics in healthcare and agriculture accelerates the spread of resistant bacteria, and efforts such as the Global Action Plan on Antimicrobial Resistance aim to address this crisis. Innovations in next-generation sequencing and bioinformatics are crucial for the early detection and prevention of AMR, allowing more targeted interventions and antibiotic stewardship programs.
Zoonoses and One Health approach [12]	∘ Emerging zoonoses with pandemic potential are prioritized in global health security, but endemic zoonoses also significantly affect low-resource settings. ∘ Many endemic zoonoses, such as rabies, brucellosis, and leptospirosis, are treatable, yet prompt diagnosis remains a challenge. ∘ The One Health approach, which integrates human, animal, and environmental health, is crucial for managing these diseases, especially in rural areas. ∘ Animal vaccination and cross-sectoral collaboration are key to strengthening fragile health systems and addressing zoonotic diseases.	The One Health approach recognizes the interconnectedness of human, animal, and environmental health. It is particularly effective in low- and middle-income countries, where zoonotic diseases cause significant human morbidity and economic loss. Rabies elimination programs in Asia and Africa have demonstrated the effectiveness of animal vaccination campaigns. Strengthening community engagement and trust in health interventions is vital for ensuring long-term success.
Climate change and health [13]	∘ Climate change disproportionately affects the poorest populations, with direct impacts like reduced food yields and storm surges and indirect effects on water, agriculture, and urban planning. ∘ Adaptation efforts are gaining attention, particularly in developing regions where climate impacts are strongest and response mechanisms are weakest. ∘ Global institutions like the World Bank and the United Nations are increasing funding for climate adaptation strategies, focusing on local vulnerabilities and community needs.	The Lancet Countdown on health and climate change highlights that vulnerable populations in low-income regions face heightened risks from climate-related health issues. Adaptation strategies, such as climate-resilient agriculture and improved water management, are key to mitigating these impacts. Supporting these efforts through funding from the Green Climate Fund and other global entities is crucial.
Non-communicable diseases (NCDs) and regenerative agriculture (RA) [14]	∘ NCDs, including metabolic syndrome and cardiovascular diseases, are rising globally and present major health and economic challenges. ∘ Regenerative agriculture (RA) practices, which emphasize soil health, offer potential health benefits by improving the quality of plant- and animal-derived foods. ∘ RA-produced foods are superior in quality and nutritional value compared with those from industrial agriculture (IA), promoting human health and environmental sustainability.	The growing incidence of NCDs has led to reevaluating dietary practices, with studies showing that plant-based diets can help prevent and manage these diseases. RA improves nutrient density in food, by promoting biodiversity and soil health, making it a promising strategy for combating NCDs. Moreover, RA helps to sequestrate carbon and promote ecosystem resilience, aligning with global goals for sustainability.
Traditional medicine and modern innovations: phytonanoparticles [15]	∘ Natural remedies remain the primary healthcare option for 80–85% of the global population, especially in developing regions. ∘ Biogenic phytonanoparticles, produced via green chemistry from metals like copper and silver, represent a sustainable and innovative therapeutic option. ∘ These plant-based nanomaterials are effective in areas like antioxidant therapies and antiaging applications, combining traditional wisdom with modern scientific advances.	The rise of nanotechnology has introduced new possibilities in medicine. Notably, the use of phytonanoparticles offers enhanced bioavailability and targeted delivery. By merging traditional knowledge of herbal medicine with modern nanotechnology, researchers can develop environmentally sustainable and scientifically advanced therapies. This approach is also being explored for its potential in treating chronic diseases like cancer and neurodegenerative disorders.
Lifestyle factors and non-alcoholic fatty liver disease (NAFLD) [16]	∘ Irregular eating patterns and disrupted sleep, commonly seen in modern lifestyles, contribute to the development of NAFLD. ∘ Circadian rhythm disruptions, such as social jetlag, are linked to negative metabolic outcomes and exacerbate the progression of NAFLD. ∘ Chrononutrition strategies, like intermittent fasting, show promise in aligning dietary habits with biological rhythms to manage NAFLD, though more research is needed.	NAFLD is closely associated with lifestyle factors such as poor diet, physical inactivity, and sleep disturbances. Chrononutrition, which focuses on eating in synchronicity with the body's internal clock, has shown the potential to improve metabolic health and mitigate the effects of NAFLD. Early clinical trials suggest that intermittent fasting can help regulate circadian rhythms, improve liver function, and reduce fat accumulation.

Table 2 shows the details of each category. By categorizing the findings into these six categories, the key aspects of each are highlighted and expanded with appropriate references to underline their significance in current global health research. Solutions are also suggested to give a more scientific approach to the most important global health and human well-being challenges in the future of humankind.

In summary, the results highlight key global health and human well-being challenges, including antimicrobial resistance (AMR), zoonotic diseases, climate change, non-communicable diseases (NCDs), and lifestyle-related disorders like non-alcoholic fatty liver disease (NAFLD). The outcome emphasizes the need for innovative, sustainable solutions such as the One Health approach to tackle zoonoses, regenerative agriculture (RA) to improve food quality and environmental sustainability, and the advancement of bioinformatics for real-time detection of AMR. Traditional remedies combined with modern science, such as biogenic phytonanoparticles, are highlighted as promising avenues for enhancing healthcare, alongside lifestyle interventions like chrononutrition to manage metabolic diseases. The overarching message of the included papers advocates for the integration of environmental, social, and technological solutions to promote global health and human well-being.

## 4. Discussion

This study presents updated challenges and opportunities in global health and human well-being, focusing on antimicrobial resistance (AMR), zoonotic diseases, and the One Health approach. While the findings emphasize existing global health issues, focusing only on global health and human well-being, the practical application of solutions varies by region due to differing infrastructures and resources. Although the One Health approach seems ideal for low-income countries, implementation barriers due to governance, healthcare systems, and cultural practices are likely/possible.

AMR is a growing global health threat that, if left unaddressed, could result in another pandemic. Despite various control/containment efforts, global AMR trends continue to rise. The primary causes include the misuse and overuse of antibiotics in healthcare and agriculture, bacterial evolution, mutations, and the transfer of resistant genes [11],[18]. This issue complicates treatment protocols, challenges the efficacy of existing antibiotics, and increases healthcare costs due to increased hospitalizations and drug use. A literature review of studies published after 2012, using databases like Scopus, PubMed, and Google Scholar, revealed that AMR's impacts span three levels: patient, healthcare, and economic. While there are clear knowledge gaps and areas for improvement, no effective solution has yet emerged to halt the growing AMR crisis [18].

Advanced DNA sequencing and bioinformatics have improved real-time AMR detection, making this area vital in future healthcare planning [19]. However, these technologies are expensive and not easily accessible in low-income regions. The advancements in DNA sequencing and bioinformatics mentioned as solutions for the real-time detection of AMR are undeniably promising. However, these technologies are costly and require substantial infrastructure, which may not be available in low- and middle-income countries. The assumption that these advancements can be easily adopted globally underestimates the financial and technological constraints many regions face. Global health solutions, affordable and scalable technologies, and/or capacity-building tailored to local contexts and informed by regional data are crucial to combating AMR [20],[21].

Zoonotic diseases, which account for over 75% of emerging pathogens, pose significant health and economic risks. These diseases account for billions of cases and deaths globally each year, posing enormous health and financial challenges. In the last decade alone, zoonotic diseases have caused over $20 billion in direct costs and $200 billion in indirect costs [22]. Human activities, the rapid growth of livestock farming, urbanization, global trade, and AMR, coupled with climate change and environmental degradation, have increased the risk of zoonotic diseases' spread and worsened disease transmission by disrupting ecosystems and increasing interactions among wildlife, domestic animals, and humans [23].

Foodborne pathogens, worsened by changes in food production, globalization, and population growth, cause millions of illnesses and deaths annually. Major zoonotic outbreaks in recent decades include Ebola, COVID-19, avian influenza, and rabies, all of which have had devastating public health and economic impacts [24],[25]. These outbreaks highlight the need for a coordinated One Health approach and a multidisciplinary approach to food safety and disease prevention.

One Health is an interdisciplinary and collaborative approach that aims to ensure optimal health by recognizing the connections among the environment, humans, animals, and plants [12],[25]. Operating at the global, regional, national, and local levels, it addresses health threats at the human–animal–environment interface to achieve sustainable public and animal health, food security, and ecosystem balance. One Health mobilizes multiple disciplines, sectors, and communities to work together to tackle global challenges like climate change. It fosters cross-sector collaboration in policy, programs, and legislation to promote collective action for better health outcomes [25]–[28]. However, implementing One Health strategies, especially in resource-limited settings, remains challenging and requires substantial investments in education, infrastructure, and cross-sector collaboration [21],[29].

Diseases like rabies, leptospirosis, and brucellosis severely affect human populations and livestock. The One Health approach has been advocated as a preventive measure. Vaccination of animals, for instance, benefits both human and animal populations, improving health outcomes and strengthening local health systems [30]. However, despite its potential, implementing the One Health strategy, particularly in low-income countries, remains limited, and more research is needed to understand its effectiveness in controlling zoonoses.

Building trust, fostering cross-sector collaboration, and integrating human, animal, and environmental health efforts require significant investments into education, infrastructure, and human resources. These factors increase the complexity of implementing the One Health approach, especially in regions with a weak or non-existent healthcare infrastructure [21]. Future analysis should include a discussion of the logistical, financial, and cultural challenges of rolling out One Health interventions, especially in rural and underserved areas.

Human-induced climate change disproportionately affects the world's poorest populations, particularly through impacts on food security, storm surges, and water systems. These direct and indirect pathways complicate health interventions and make vulnerable communities more prone to negative outcomes [31]. Funding and support for climate adaptation from international organizations like the UN and the World Bank are increasing; however, the success of these initiatives depends on their alignment with local vulnerabilities and needs [32]. Current scientific evidence underscores the significant global health impacts of climate change. While some uncertainty about its extent persists, this uncertainty is gradually decreasing, and a strong consensus exists, with 97% of climatologists agreeing that human activities, particularly the burning of fossil fuels and deforestation, are major drivers of climate change [33]. However, questions remain regarding the specific risks, vulnerabilities, and the most effective policies for adaptation (to minimize negative effects) and mitigation (to reduce heat-trapping emissions) [34].

NCDs, such as diabetes, cardiovascular diseases, and metabolic disorders, are rising globally, with diet playing a central role in both the development and prevention of these conditions. Several studies underscore the potential of RA, which prioritizes soil health and produces more nutrient-rich foods, in mitigating these diseases [35],[36]. RA practices improve food quality and reduce the environmental impact of agriculture, offering a holistic solution to both health and ecological challenges [37].

However, while studies highlight RA as a solution for human health and environmental sustainability, the evidence supporting RA's superiority over conventional agriculture in mitigating NCDs is still emerging. The current body of literature does not unequivocally demonstrate that RA-produced foods consistently offer higher nutritional value, nor that RA alone can mitigate NCDs without broader dietary and systemic changes. Nevertheless, RA is a promising area for further research, acknowledging the limitations of current evidence and the need for more rigorous studies to validate these claims.

The gut microbiome plays an essential role in various diseases, including metabolic syndrome, liver disorders, inflammatory bowel disease, and colon cancer. Changes in diet can positively impact the microbiome, fostering a symbiotic relationship between microbes and the human body [38]. This points to the significant potential of dietary modifications as a strategy for disease prevention and management. The parallels between soil health in agriculture and the human gut microbiome further highlight the interconnectedness of environmental and human health [39].

Many studies emphasize the role of technological advancements (e.g., bioinformatics, DNA sequencing) in solving health challenges [40],[41]. While these tools are undeniably valuable, they are not standalone solutions. Complex global health challenges, like AMR, zoonotic diseases, and climate change, require a mix of social, political, economic, and technological solutions. There is an over-reliance on technological innovations without an equal emphasis on policy changes, healthcare reforms, or public health strategies, which are also critical for long-term success. Future policies should better integrate non-technological approaches, such as strengthening healthcare systems, enhancing regulatory frameworks, and fostering global collaboration [21],[42].

The use of traditional natural remedies remains a significant aspect of healthcare for many populations globally. Biogenic phytonanoparticles, created through green chemistry techniques, have emerged as a promising avenue in therapeutic interventions. They offer non-toxic, sustainable, and cost-effective solutions and are aligned with environmental and health considerations [43]. These innovations reflect the blending of traditional knowledge with modern technology, marking new frontiers in global healthcare [44]. The use of biogenic phytonanoparticles, however, does not fully address the challenges of integrating these traditional remedies with modern scientific practices. Issues such as regulatory oversight, standardization, and clinical validation remain significant barriers to bringing these treatments into mainstream healthcare. Future discussions should address the need for rigorous clinical trials, international regulatory frameworks, and quality control in the use of phytonanoparticles.

Lifestyle factors, including irregular eating patterns and disrupted circadian rhythms, are key drivers of conditions such as non-alcoholic fatty liver disease (NAFLD). The misalignment between circadian and social clocks, termed “social jetlag,” negatively affects metabolic health. Chrononutrition strategies, such as intermittent fasting and time-restricted feeding, have shown promise in aligning lifestyle behaviors with biological rhythms, although more research is needed [45]. Nevertheless, the potential use of chrononutrition to address NAFLD lacks specificity in addressing the practical challenges of adopting these dietary strategies. Factors such as socioeconomic barriers, cultural preferences, and individual metabolic differences can significantly affect the success of chrononutrition. A more thorough analysis would consider how to overcome socioeconomic and cultural barriers in implementing these dietary interventions.

Promoting positive social norms and behavioral changes, such as adherence to responsible antibiotic use or shifts in dietary practices, is not straightforward. Many factors influence behavioral change, including leadership, socioeconomic status, education, cultural beliefs, and healthcare's accessibility [6],[46]. Assuming that awareness campaigns and promoting social norms will lead to significant behavioral changes is often not the case. Such an assumption simplifies the challenges of influencing large-scale behavioral change, and overlooks social and cultural barriers, while it should incorporate the complexity of behavioral interventions, including the need for multifaceted, long-term approaches that combine education, regulation, and community involvement [47].

## 5. Limitations

While the results of this study provide a promising outlook on addressing global health challenges, the broad application of the discussed concepts does not fully capture regional, cultural, and economic variations. These concepts could be strengthened by incorporating a deeper analysis of local constraints, technological access, behavioral complexities, and the need for rigorous scientific validation in areas like regenerative agriculture and traditional remedies. More specific data tailored to local health systems would strengthen the analysis. Future discussions should focus more on region-specific challenges and solutions to avoid overgeneralizing global health strategies. The focus on technological and scientific advancements, while important, should be balanced with discussions of social, cultural, and policy-based solutions that are necessary for long-term, sustainable progress.

The number of papers included, including narrative reviews, did not allow statistically presentable data. However, this paper attempts to provide detailed overviews of global health and human well-being in a comprehensive discussion examining the challenges of these two areas in combination as one of the study's objectives to offer a holistic understanding of how these concepts are interrelated, examining how both topics in unison could help identify strategies that address the root causes of these challenges, enabling a more comprehensive and effective approach to improving the overall health and well-being of populations worldwide. There are no upper or lower limits to the number of studies obtained in a systematic search. A range of 5 to 20 studies appears to be common. In this study, the final number of included articles was seven, which is within the accepted range. Some other studies have reported empty views [48],[49]. Our inclusion and exclusion criteria were clearly defined, and the protocol was pre-registered to guarantee transparency. In this context, as Gray cites in his study, “If the results are returning too many—or too few—studies, that is not a reason to change the methodology to make the numbers work in your favor” (page 29) [48].

It is also worth noting that a number of the papers identified in the initial search conflated the term global health with planetary health and/or environmental health, without specifically focusing on the health of human populations. Scholars need to be mindful of their definitions of terms such as these and be clear about which terms are correct for their particular focus. In this study, we carefully checked for synonyms of well-being, avoiding words associated with other topics (e.g., planetary health), focusing only on global health and human well-being. Despite this, our search included studies that discuss planetary health, among others. This shows the interconnectedness of these subjects, even though our focus was set on our selected keywords.

## 6. Conclusions

This study highlights the need for scalable, affordable solutions that integrate global health, environmental sustainability, and local realities to address the challenges of AMR, zoonoses, and climate-related health issues. The study highlights the importance of adopting an integrated, interdisciplinary approach to global health, drawing on insights from medical microbiology, agriculture, environmental science, and traditional remedies. Whether through addressing AMR with bioinformatics advancements, promoting One Health strategies, or fostering sustainable food systems through regenerative agriculture, global health solutions must be holistic and inclusive. These findings align with broader health equity goals and emphasize the interconnectedness of humans, animals, and environments for global health and human well-being.

Moreover, lifestyle behaviors like poor diet, inactivity, inadequate sleep, and chronic stress significantly affect well-being, contributing to NCDs such as heart disease, diabetes, and mental health disorders. While these factors disrupt physical and mental health, balanced nutrition, regular exercise, stress management, and quality sleep can improve health and enhance quality of life.

## Use of AI tools declaration

The authors declare they have not used artificial intelligence (AI) tools in the creation of this article.
